# The fetal sheep lung does not respond to cortisol infusion during the late canalicular phase of development

**DOI:** 10.1002/phy2.130

**Published:** 2013-11-11

**Authors:** Erin V McGillick, Sandra Orgeig, I Caroline McMillen, Janna L Morrison

**Affiliations:** 1 Early Origins of Adult Health Research Group, School of Pharmacy & Medical Sciences, Sansom Institute for Health Research, University of South AustraliaAdelaide, South Australia, Australia, 5001; 2 Molecular & Evolutionary Physiology of the Lung Laboratory, School of Pharmacy & Medical Sciences, Sansom Institute for Health Research, University of South AustraliaAdelaide, South Australia, Australia, 5001

**Keywords:** Glucocorticoid, liquid reabsorption, lung, preterm, surfactant

## Abstract

The prepartum surge in plasma cortisol concentrations in humans and sheep promotes fetal lung and surfactant system maturation in the support of air breathing after birth. This physiological process has been used to enhance lung maturation in the preterm fetus using maternal administration of betamethasone in the clinical setting in fetuses as young as 24 weeks gestation (term = 40 weeks). Here, we have investigated the impact of fetal intravenous cortisol infusion during the canalicular phase of lung development (from 109- to 116-days gestation, term = 150 ± 3 days) on the expression of genes regulating glucocorticoid (GC) activity, lung liquid reabsorption, and surfactant maturation in the very preterm sheep fetus and compared this to their expression near term. Cortisol infusion had no impact on mRNA expression of the corticosteroid receptors (GC receptor and mineralocorticoid receptor) or *HSD11B-2*, however, there was increased expression of *HSD11B-1* in the fetal lung. Despite this, cortisol infusion had no effect on the expression of genes involved in lung sodium (epithelial sodium channel -α, -β, or -γ subunits and sodium–potassium ATPase-β1 subunit) or water (aquaporin 1, 3, and 5) reabsorption when compared to the level of expression during exposure to the normal prepartum cortisol surge. Furthermore, in comparison to late gestation, cortisol infusion does not increase mRNA expression of surfactant proteins (*SFTP-A*, *-B*, and *-C*) or the number of SFTP-B-positive cells present in the alveolar epithelium, the cells that produce pulmonary surfactant. These data suggest that there may be an age before which the lung is unable to respond biochemically to an increase in fetal plasma cortisol concentrations.

## Introduction

Before birth the placenta performs the role of gas exchange for the developing fetus (Longo and Reynolds 2010). Successful transition from the intrauterine to the extrauterine environment depends on the ability of the neonatal lungs to assume this vital function at birth. The fetal lung develops as a fluid-filled sac (Hooper and Harding 1995) and it is the reabsorption of the liquid which has bathed the alveolar epithelium throughout gestation that is an important determinant of the successful transition to air breathing at birth. Lung liquid reabsorption is driven by an osmotic gradient generated by transepithelial sodium movement within the lung. The process of sodium transport from the lung lumen is controlled by the amiloride-sensitive voltage-gated epithelial sodium channel (SCNN1), comprised of -α (SCNN1-A), -β (SCNN1-B) and -γ (SCNN1-G) subunits, which is present on the luminal surface of the pulmonary epithelium (Snyder 2005) and sodium–potassium active transport pumps (ATP1), comprised of catalytic α (ATP1A1) and glycolytic β (ATP1B1) subunits, which are present on the basal surface of the pulmonary epithelium (Ewart and Klip 1995). The rapid loss of water from the lung lumen in response to the net movement of sodium is regulated by differential expression of the aquaporin (AQP; subtypes 1, 3, 4 and 5) family of transmembrane channel proteins in the lung (Liu and Wintour 2005).

With the first breath, an air–liquid interface is generated within the alveoli, as the lung fluid is reduced to a thin layer, this aqueous hypophase creates high surface tension. This makes inflation difficult and as a result, a stabilizing force within the lung that prevents alveolar collapse throughout the breathing cycle is required. Pulmonary surfactant is secreted from type II alveolar epithelial cells (AEC) lining the alveoli. Surfactant is a complex mixture of lipids and surfactant proteins (SFTP), which primarily functions to reduce surface tension (Creuwels et al. 1997). The surfactant lipid component forms a stable surface film at the air-liquid interface to reduce and vary the surface tension with changing lung volume (Goerke and Clements 1985). In addition, SFTP-B and -C play important roles in regulating surface tension as they function cooperatively to promote adsorption and spreading of surfactant lipids (Hawgood et al. 1998; Johansson 1998). SFTP-A and -D play important roles in innate immunity within the lung (LeVine and Whitsett 2001; Haagsman et al. 2008). Type II AECs, which produce the components of surfactant, appear from mid-gestation during the canalicular phase of lung development (humans, ∼16–25 weeks, term = 40 weeks; sheep, ∼80–120 days, term = 150 ± 3 days) (Harding and Bocking 2001).

Maturation of the surfactant system occurs in the saccular phase of lung development in late gestation (humans, from ∼25 weeks to term; sheep, from ∼120 days to term), and is largely regulated by the prepartum rise in the concentration of plasma cortisol, the endogenous glucocorticoid (GC) (Silver and Fowden 1988; Tan et al. 1999; Bolt et al. 2001). In addition, GCs regulate the expression of genes controlling lung liquid reabsorption both in vivo and in vitro (Champigny et al. 1994; Dagenais et al. 2001; Mustafa et al. 2004; Jesse et al. 2009). Intracellular GC concentrations are regulated by two isoforms of the 11β-hydroxysteroid dehydrogenase (HSD11B) enzyme, with HSD11B-1 generating cortisol from inactive cortisone, while HSD11B-2 functions to interconvert active cortisol to cortisone (Tomlinson and Stewart 2001). GCs mediate their effects through the intracellular glucocorticoid receptor (NR3C1). Once bound, the GC-NR3C1 complex binds to glucocorticoid response elements (GRE) present on target genes to induce their expression. The SFTP promoters do not contain a GRE, however, their expression is regulated indirectly by GC through transcription factors that have a GRE and bind to the SFTP promoter to increase gene expression (Reichardt et al. 1998; Mendelson 2000). It has been suggested that this mechanism involves transcription factors such as thyroid transcription factor-1 (NKX2-1) and cofactors such as GATA-binding protein-6 (GATA6) (Stahlman et al. 1996; Mendelson 2000). In addition to the SFTPs, there is evidence to suggest that indirect GC activity is also a mechanism by which GCs regulate expression of genes involved in lung liquid reabsorption (Bremner et al. 2002). GC action within the fetal lung is also mediated through the action of the mineralocorticoid receptor (NR3C2), which is known to play a role in normal lung development and lung liquid reabsorption (Keller-Wood et al. 2009). It is not known if these genes respond when plasma cortisol concentrations reach a threshold level or if their expression is dependent on the structural development of the lung, including the number of type II AECs.

In this study, we have evaluated the effectiveness of fetal intravenous cortisol infusion to influence expression of genes regulating lung liquid reabsorption and SFTPs in the very preterm sheep fetus during the late canalicular phase of lung development, equivalent to ∼23–24 weeks gestation in humans, compared to age-matched controls and fetuses during the prepartum cortisol surge in late gestation.

## Methods

All procedures were approved by the University of Adelaide Animal Ethics Committee. Tissues used in this study were available from a series of fetal sheep studies and data on neuroendocrine function from some of these animals have been published previously (Warnes et al. 1998; Ross et al. 2000).

### Animals and surgery

Twenty-nine pregnant Border-Leicester × Merino and Merino ewes were used in this study. The ewes were housed in individual pens in animal holding rooms, with a 12:12 h light/dark cycle, and fed once daily with water provided ad libitum. At 103- or 119-days gestation, general anesthesia was induced in the ewe with an intravenous injection of sodium thiopentone (1.25 g, Pentothal; Rhone Merieux, Pinkenba, Qld, Australia) and maintained with 2.5–4% halothane inhalation anesthetic (Fluothane; ICI, Melbourne, Vic, Australia) in oxygen. Vascular catheters were implanted in the jugular vein of the ewe, a carotid artery, and jugular vein of the fetus, and in the amniotic cavity as previously described (Ross et al. 2000; Muhlhausler et al. 2005). Ewes received an intramuscular injection of antibiotics (3.5 mL of Norocillin [Norbrook Laboratories Ltd., Gisborne, Australia] and 2 mL of 125 mg/mL Dihydrostreptomycin in sterile saline [Sigma, St Louis, MO]) for 3 days following surgery. Antibiotics (500 mg; sodium ampicillin; Commonwealth Serum Laboratories, Melbourne, Vic, Australia) were administered intra-amniotically to all fetal sheep daily for 4 days postoperatively. Ewes were allowed at least 4 days to recover from surgery prior to the experimental protocol.

### Infusion regime

Cortisol (hydrocortisone succinate, Solucortef, 2–3 mg in 4.4 mL saline/24 h) was infused intravenously into the fetus from 109- to 116-days gestation (*n* = 9). Age-matched controls received either saline infusion from 109- to 116-days gestation (*n* = 4) or no infusion (*n* = 4). This infusion protocol has previously been shown to increase fetal plasma cortisol concentrations (saline-infused, 1.6 ± 0.1 nmol/L; cortisol-infused, 39.3 ± 2.8 nmol/L [Ross et al. 2000; Warnes et al. 1998]). Late gestation fetuses received saline infusion from 130- to 140-days gestation (*n* = 12) and plasma cortisol concentration at this gestational age is 5–10 nmol/L (Phillips et al. 1996; Morrison et al. 2007; Orgeig et al. 2010).

### Fetal arterial blood gas measurements

Fetal arterial blood samples (1 mL) were collected daily to measure PaO_2_, PaCO_2_, pH, oxygen saturation (S_a_O_2_) and hemoglobin (Hb) using an ABL 550 analyzer (Radiometer Pacific Pty Ltd, Australia) and temperature corrected to 39°C.

### Tissue collection

At 116 ± 1 day (*n* = 17) or 140 ± 1 day (*n* = 12) gestation, ewes were humanely killed with an intravenous overdose of sodium pentobarbitone (Virbac Pty Ltd, Peakhurst, NSW, Australia). Fetal sheep were delivered by hysterectomy, weighed, and humanely killed. The fetal lungs were removed, weighed, snap frozen in liquid nitrogen, and stored at −80°C for subsequent gene expression analysis. In addition, a section of lung tissue was fixed in 4% paraformaldehyde for subsequent immunohistochemical analysis.

### Quantification of mRNA transcripts within the fetal lung

#### Total RNA extraction

Total RNA was extracted from 22 lung samples (∼50 mg) using Invitrogen Trizol Reagent Solution (Invitrogen Life Technologies, Carlsbad, CA) as per the manufacturer's guidelines and Qiagen RNeasy purification columns (Qiagen Pty Ltd., Doncaster, Vic, Australia) (Gentili et al. 2009; Soo et al. 2012). Total RNA integrity from all extracted tissue samples was assessed by running all samples (116-day saline-infused = 6; 116-day cortisol-infused = 6; 140-day saline-infused = 10) on an agarose gel stained with ethidium bromide. Total RNA was quantified by spectrophotometric measurements at 260 and 280 nm and checked for protein and DNA contamination. cDNA was synthesized using Superscript III First Strand Synthesis System (Invitrogen) using 2 μg of total RNA, random hexamers, dNTP, DTT and Superscript III in a final volume of 20 μL as per the manufacturer's guidelines. Controls containing either no RNA transcript (no template control – NTC) or no Superscript III (no amplification control, NAC) were used to test for reagent contamination and genomic DNA contamination, respectively.

#### Quantitative real-time RT-PCR

Initially, the geNorm component of qbase^plus^ 2.0 software (Biogazelle, Zwijnaarde, Belgium) was used to determine the most stable reference genes from a panel of candidate genes (Vandesompele et al. 2002) and the minimum number of reference genes required to calculate a stable normalization factor as previously described (Soo et al. 2012). The gene expression of GC regulatory genes (*HSD11B-1*, *HSD11B-2*, *NR3C1*, *NR3C2, NKX2-1*, and *GATA6*), genes regulating lung liquid reabsorption (*SCNN1-A*, *SCNN1-B*, *SCNN1-G*, *ATP1A1* (Keller-Wood et al. 2009), *ATP1B1*, *AQP1*, *AQP3*, *AQP4*, and *AQP5*), surfactant proteins (*SFTP-A*, *-B*, *-C* [Orgeig et al. 2010], and *-D*), and reference genes (β-actin (*ACTB*), peptidylprolyl isomerase A (*PPIA*) (Passmore et al. 2009), and tyrosine 3-monooxygenase (*YWHAZ*)) were measured by qRT-PCR. Previously published (Keller-Wood et al. 2005; Passmore et al. 2009; Orgeig et al. 2010) or specifically designed (Table 1) primer sets were validated and optimized as previously described (Orgeig et al. 2010). mRNA transcripts in all fetal lung samples were measured by qRT-PCR using Fast SYBR® Green Master Mix (Applied Biosystems, Foster City, CA) in a final volume of 6 μL on a ViiA7 Fast Real-time PCR system (Applied Biosystems). Each qRT-PCR well contained 3 μL Fast SYBR Green Master Mix (2×), 2 μL of forward and reverse primer mixed with H_2_O to obtain final primer concentrations (Table 1) and 1 μL of diluted relevant cDNA. Primers were validated to generate a single transcript as confirmed by the presence of an individual double stranded DNA product of the correct size and sequence. Controls for each primer set containing no cDNA were included on each plate to test for reagent contamination (NTC). Melt curve/dissociation curves were also run to check for nonspecific product formation. Amplification efficiency reactions were performed on five triplicate serial dilutions of cDNA template for each primer set. Amplification efficiencies were determined from the slope of a plot of C_t_ (defined as the threshold cycle with the lowest significant increase in fluorescence) against the log of the cDNA template concentration (ranging from 1 to 100 ng). C_t_ values were in the linear amplification range for all genes. Each sample was run in triplicate for target genes and reference genes. The reactions were quantitated by setting the threshold within the exponential growth phase of the amplification curve and obtaining corresponding C_t_ values. The abundance of each transcript relative to the abundance of stable housekeeping genes (Hellemans et al. 2007) was calculated using DataAssist 3.0 analysis software (Applied Biosystems) and expressed as mean normalized expression (Soo et al. 2012).

**Table 1:** qRT-PCR primer sequences and final primer concentrations for target and housekeeping genes

Primer name	Sequence 5′→3′	Primer conc (μmol/L)	Accession no.
HSD11B-1			NM_001009395.1
Forward	GCGCCAGATCCCTGTCTGAT	0.90	
Reverse	AGCGGGATACCACCTTCTTT	0.90	
HSD11B-2			NM_001009460.1
Forward	GAGACATGCCGTTTCCATGC	0.45	
Reverse	TGATGCTGACCTTGACACCC	0.45	
NR3C1			NM_001114186.1
Forward	ACTGCCCCAAGTGAAAACAGA	0.90	
Reverse	ATGAACAGAAATGGCAGACATTTTATT	0.90	
NR3C2			AF349768.1
Forward	ATGACAGCTCCAAACCAAACACGG	0.90	
Reverse	AAATCCTGGAAGTACCTTCGCCCA	0.90	
NKX2-1			FJ177515
Forward	ACACAAAGACCAAACTGCTGGACG	0.90	
Reverse	GCGTGGGAAACCCATTTGAATCAC	0.90	
GATA6			DQ126151
Forward	TCTACAGCAAGATGAACGGCCTCA	0.90	
Reverse	TAGAGTCCACAGGCATTGCACACA	0.90	
SCNN1-A			AF232715.1
Forward	ACGACAAGAACAGCTCCAACCTCT	0.90	
Reverse	GCCGCAGATTAAAGCCAGCATCAT	0.90	
SCNN1-B			AF065146.1
Forward	AGTGGTTCTGGACCTGTTTGAGGA	0.45	
Reverse	CATGTGGTTCCATTGTGGCTGCAT	0.45	
SCNN1-G			AF250862.1
Forward	TCGTGCTTCCAGGCAAAGATGGTA	0.45	
Reverse	TTAAAGCTGCAGGCTTCCTTGCAC	0.45	
ATP1B1			NM_001009796
Forward	TGCCTTTCGTCCTAACGATCCCAA	0.45	
Reverse	CTGGGCACATTGCCACAATCTTCA	0.45	
AQP1			NM_001009194.1
Forward	AAAGTGTCACTGGCCTTTGGGTTG	0.45	
Reverse	ATGTACATGATGGCCCGGAGGATA	0.45	
AQP3			AF123316.1
Forward	TCACTTGAACCCTGCTGTGACCTT	0.05	
Reverse	ACCCGAAGATAATTCCAGCACCCA	0.05	
AQP4			NM_001009279
Forward	TGGGAAATTGGGAGAACCACTGGA	0.45	
Reverse	GGCAGCTTTGCTGAAGGCTTCTTT	0.45	
AQP5			NM_001009273
Forward	CAATCTGGCTGTCAATGCGCTCAA	0.45	
Reverse	AGTCAGTGGAAGAGAAGACGCACA	0.45	
SFTP-D			AJ133002.1
Forward	GGCCACAGCCCAGAACAA	0.3	
Reverse	AAGTACCCTCCTTCCTGGTATCG	0.3	
YWAHZ			AY970970
Forward	TGTAGGAGCCCGTAGGTCATCT	0.45	
Reverse	TTCTCTCTGTATTCTCGAGCCATCT	0.45	

Accession numbers refer to the published cDNA sequences from which the primer sequences were designed.

### Quantification of type II AECs within the fetal lung

#### SFTP-B immunoreactivity to identify mature AECs

Immunohistochemistry was performed in a subset of animals (116-day saline-infused = 2; 116-day cortisol-infused = 4; 140-day saline-infused = 5) using a monoclonal antibody to SFTP-B (produced by Dr Y. Suzuki, Kyoto University, Japan and kindly donated by F. Possmayer, University of Western Ontario, Canada), staining of which is restricted to type II AECs in the alveolar epithelium and Clara cells in the bronchiolar epithelium (Weaver 1998; Lin et al. 1999). Paraformaldehyde-fixed, paraffin-processed lung tissue sections of 7-μm thickness were deparaffinized and rehydrated before endogenous peroxide solution activity was blocked, and followed by antigen retrieval. Slides were incubated overnight with the aforementioned SFTP-B antibody (1:1000) at 4°C. Negative control slides were performed in parallel with test slides. A Histostain-Plus broad spectrum kit (Zymed Laboratories Inc., South San Francisco, CA) was utilized with HRP and 3,3-diaminobenzidine (DAB) chromagen (Metal Enhanced DAB Substrate Kit; Pierce Biotechnology, Rockford, IL) for visualization of SFTP-B-positive cells. All sections were counterstained with Mayer's Hematoxylin.

#### Quantitative assessment of SFTP-B-positive cells in the fetal lung

Sections were examined using Visiopharm new Computer Assisted Stereological Toolbox (NewCAST) software (Visiopharm, Hoersholm, Denmark). Analysis was carried out by a single trained individual who was blinded to treatment groups. Sixty counting frames (400× magnification) of the alveolar epithelium were randomly selected per section. Point counting using an unbiased counting frame with an area of 20,000 μm^2^ was used to estimate the numerical density of SFTP-B-positive cells within the fetal lung. Using the four corners of the test frame, the reference space was estimated from the points falling on lung tissue. The numerical density of SFTP-B-positive cells expressed as SFTP-B-positive cells per mm^2^ of lung tissue was obtained using the following equation (Brüel et al. 2005):


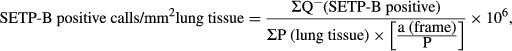


where 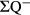 (SFTP-B Positive) represents the total number of SFTP-B-positive cells counted in all counting frames of one fetal lung tissue section; 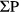 (lung tissue) represents the total number of points falling on lung tissue (i.e., the reference space); P is the number of points which were used to count the points hitting the reference space (i.e., four corners per counting frame); and *a* was the area of the counting frame. Tissue sections were photographed using a digital camera DP72 (Olympus Australia Pty. Ltd, Mt Waverley, Vic, Australia), which was connected to a BX53 Research Microscope (Olympus Australia Pty. Ltd).

### Statistical analyses

All statistical analyses were performed using Statistical Package for Social Sciences (SPSS) v20.0 (Chicago, IL). All fetal parameters and normalized mRNA expression data were analyzed using a one-way ANOVA for treatment followed by a Duncan post hoc test. The numerical density of SFTP-B-positive cells present in the alveolar epithelium was analyzed with an unpaired Student's *t*-test between 116-day cortisol and 140-day saline-infused fetuses. All data are presented as mean ± SEM. A probability level of 5% (*P* < 0.05) was considered statistically significant.

## Results

### No effect of cortisol infusion on fetal health and growth

There was no change in mean gestational Pao_2_ or Paco_2_ with age or cortisol infusion (Table 2). pH and hemoglobin content increased with age. There was a significant increase in fetal weight, crown-rump length, and lung weight as a result of age, but not cortisol infusion. Relative lung weight was not affected by age or cortisol infusion (Table 3).

**Table 2:** Mean blood gas values for 116-day saline and cortisol-infused fetuses and 140-day saline-infused fetuses throughout the infusion period

	116-day		140-day
		
	Saline-infused (*n* = 8)	Cortisol-infused (*n* = 9)	Saline-infused (*n* = 12)
PaO_2_ (mmHg)	21.5 ± 0.9	22.4 ± 0.9	22.6 ± 0.5
PaCO_2_ (mmHg)	49.6 ± 1.0	47.7 ± 0.5	49.6 ± 0.9
pH	7.335 ± 0.012^a^	7.352 ± 0.008^a^	7.379 ± 0.006^b^
Hb (g/dL)	8.8 ± 0.1^a^	9.0 ± 0.4^a^	10.1 ± 0.3^b^

Data are expressed as mean ± SEM. Data were analyzed by one-way ANOVA followed by Duncan post hoc test. *P* < 0.05 was considered statistically significant; columns with different letters are significantly different from each other. PaO_2_, arterial partial pressure of oxygen; PaCO_2_, arterial partial pressure of carbon dioxide; Hb, hemoglobin.

**Table 3:** Effect of fetal intravenous saline or cortisol infusion on fetal and lung growth

	116-day		140-day
		
	Saline-infused (*n* = 8)	Cortisol-infused (*n* = 9)	Saline-infused (*n* = 12)
Gestational age at postmortem (days)	116 ± 1^a^	116 ± 1^a^	140 ± 1^b^
Fetal weight (kg)	2.17 ± 0.1^a^	2.12 ± 0.1^a^	4.81 ± 0.2^b^
Crown-rump length (cm)	44.6 ± 0.8^a^	43.1 ± 0.4^a^	58.3 ± 1.4^b^
Lung weight (g)	75.8 ± 3.5^a^	66.2 ± 3.8^a^	158.0 ± 6.7^b^
Relative lung weight (g/kg)	37.4 ± 1.9	32.0 ± 1.2	33.8 ± 1.3

Data expressed as mean ± SEM. Data were analyzed by one-way ANOVA followed by Duncan post hoc test. *P* < 0.05 was considered statistically significant; columns with different letters are significantly different from each other.

### Effect of cortisol infusion on GC availability and signaling in the fetal lung

**Figure 1:**
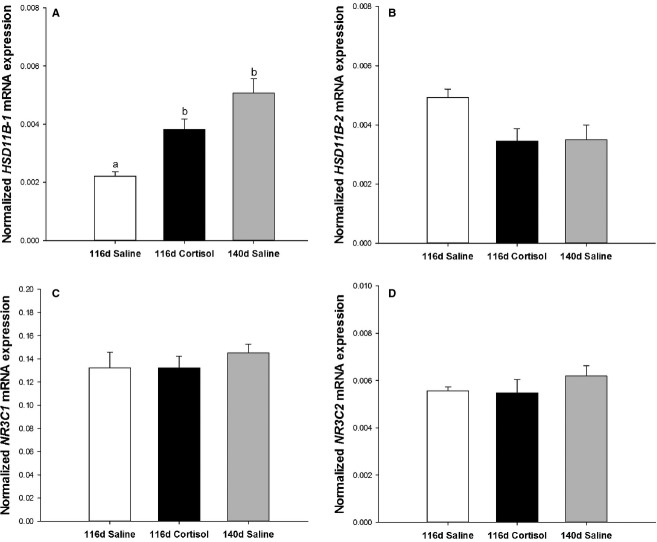
Fetal intravenous cortisol infusion increased glucocorticoid activating enzyme mRNA expression in the fetal lung. Normalized mRNA expression of 11β hydroxysteroid dehydrogenase isoform 1 (*HSD11B-1*, A) was increased with age and cortisol infusion. There was no change in normalized mRNA expression of the glucocorticoid deactivating enzyme (*HSD11B-2*, B), glucocorticoid receptor (*NR3C1*, C) or the mineralocorticoid receptor (*NR3C2*, D) with age or cortisol infusion. Data expressed as mean ± SEM. *P* < 0.05 was considered statistically significant; columns with different letters are significantly different from each other. 116-day saline-infused, open bars; 116-day cortisol-infused, black bars; 140-day saline-infused, gray bars.

There was an increase in the mRNA expression of *HSD11B-1* with both age and cortisol infusion (Fig. 1A). However, there was no effect of age or cortisol infusion on mRNA expression of *HSD11B-2, NR3C1* or *NR2C2* (Fig. 1B–D).

### Effect of cortisol infusion on the expression of genes regulating lung liquid reabsorption

**Figure 2:**
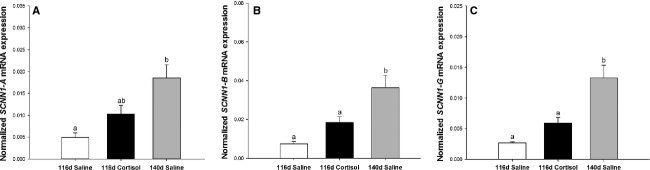
Fetal intravenous cortisol infusion from 109- to 116-days gestation had no impact on epithelial sodium channel (*SCNN1*) subunit mRNA expression compared to the late gestation fetal lung. There was an increase in normalized mRNA expression of *SCNN1-A* (A), *SCNN1-B* (B) and *SCNN1-Y* (C) subunits with age, but not cortisol infusion. Data expressed as mean ± SEM. *P* < 0.05 was considered statistically significant; columns with different letters are significantly different from each other. 116-day saline-infused, open bars; 116-day cortisol-infused, black bars; 140-day saline-infused, gray bars.

**Figure 3:**
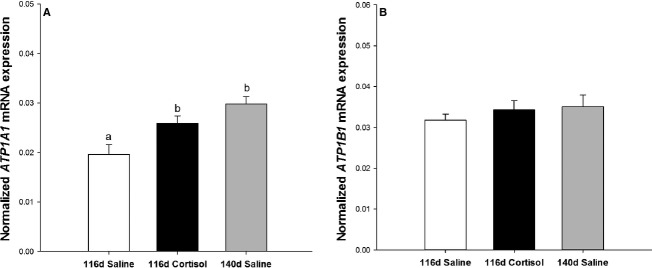
Fetal intravenous cortisol infusion increased sodium–potassium ATPase (*ATP1*) A1 pump subunit mRNA expression. There was an increase in normalized *ATP1A1* subunit mRNA expression with age and cortisol infusion (A), but no impact on the *ATP1B1* subunit (B). Data expressed as mean ± SEM. *P* < 0.05 was considered statistically significant; columns with different letters are significantly different from each other. 116-day saline-infused, open bars; 116-day cortisol-infused, black bars; 140-day saline-infused, gray bars.

**Figure 4:**
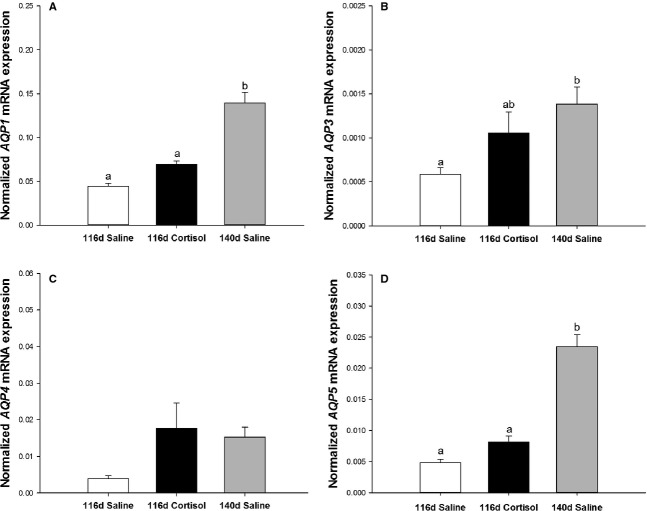
Fetal intravenous cortisol infusion did not increase expression of genes regulating water reabsorption in the fetal lung to the same extent as those observed in late gestation. There was an increase in aquaporin (*AQP*) *1*, *3*, and *5* mRNA expression with age, but not cortisol infusion (A, B, and D). *AQP4* mRNA expression was not changed with age or cortisol infusion (C). Data expressed as mean ± SEM. *P* < 0.05 was considered statistically significant; columns with different letters are significantly different from each other. 116-day saline-infused, open bars; 116-day cortisol-infused, black bars; 140-day saline-infused, gray bars.

mRNA expression of *SCNN1 -A*, *-B*, and *-G* subunits increased with age, but not cortisol infusion (Fig. 2). mRNA expression of *ATP1A1*, but not *B1*, increased with age and cortisol infusion (Fig. 3). mRNA expression of *AQP1*, *3* and *5* were increased with age, but not cortisol infusion. *AQP4* mRNA expression was not affected by age or cortisol infusion (Fig. 4).

### Cortisol infusion increased *SFTP-D* but not *SFTP-A*, *-B* or *-C* in the fetal lung

**Figure 5:**
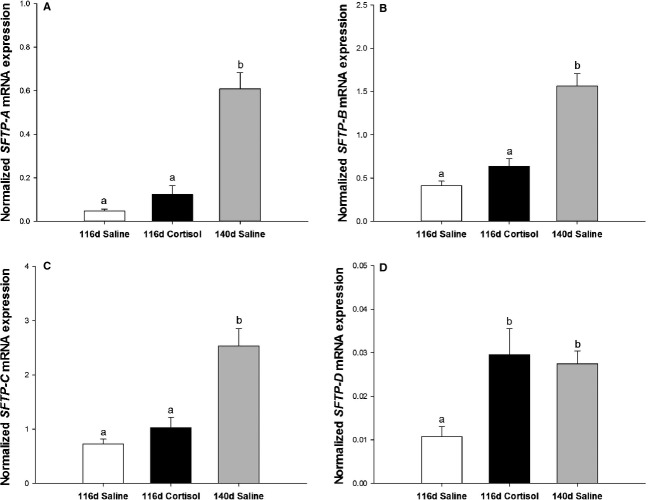
Fetal intravenous cortisol infusion does not increase surfactant protein (*SFTP*) -A, -B or -C mRNA expression in the lung compared to the late gestation fetus. There was only an increase in normalized mRNA expression of *SFTP-A* (A), *SFTP-B* (B) and *SFTP-C* (C) with age, but *SFTP-D* (D) expression was increased with both age and cortisol infusion. Data expressed as mean ± SEM. *P* < 0.05 was considered statistically significant; columns with different letters are significantly different from each other. 116-day saline-infused, open bars; 116-day cortisol-infused, black bars; 140-day saline-infused, gray bars.

**Figure 6:**
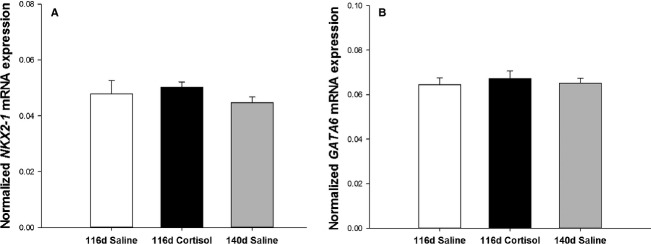
mRNA expression of transcription factor, *NKX2-1*, and its cofactor, *GATA6*, are not changed in the fetal lung with age or cortisol infusion. There was no change in normalized mRNA expression of either *NKX2-1* (A) or *GATA6* (B) with age or cortisol infusion. Data expressed as mean ± SEM. *P* < 0.05 was considered statistically significant; columns with different letters are significantly different from each other. 116-day saline-infused, open bars; 116-day cortisol-infused, black bars; 140-day saline-infused, gray bars.

*SFTP-A*, *-B*, *-C*, and *-D* mRNA expression increased with age, but only *SFTP-D* mRNA expression increased with cortisol infusion (Fig. 5). The mRNA expression of *NKX2-1*, and its cofactor, *GATA6*, were not altered by age or cortisol infusion (Fig. 6).

### Effect of cortisol infusion on the numerical density of SFTP-B-positive cells in the fetal lung

**Figure 7:**
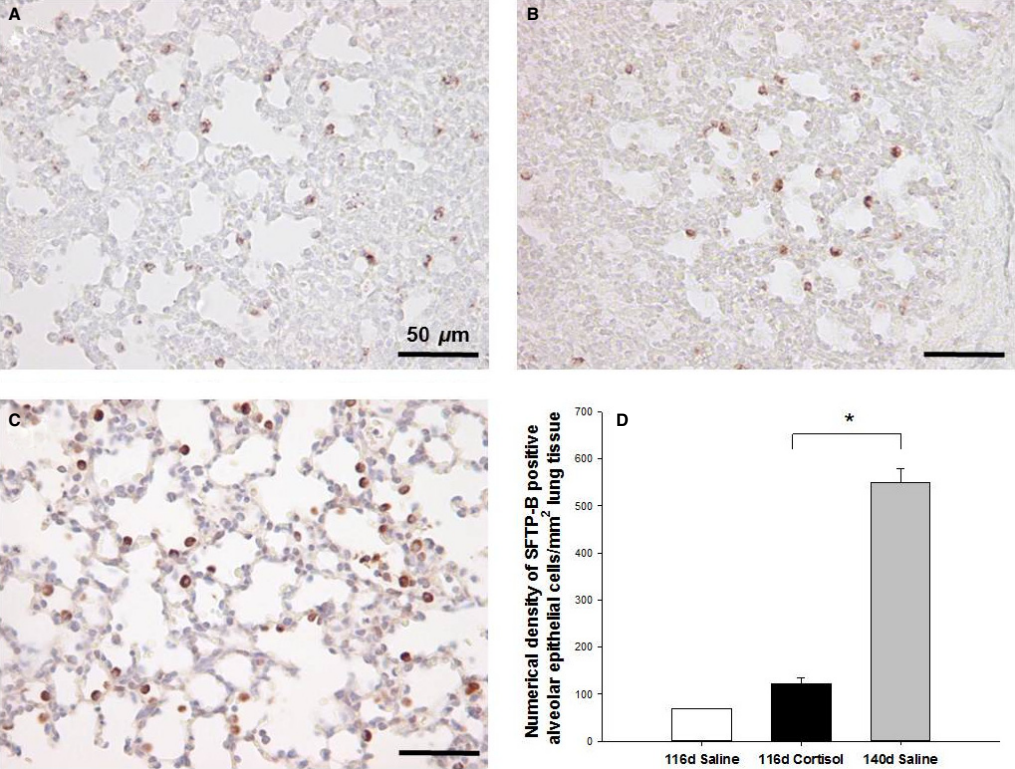
Evaluation of SFTP-B-positive alveolar epithelial cells in the fetal lung. Micrographs demonstrating SFTP-B immunoreactivity of alveolar epithelial cells in the 116-day saline-infused (A), 116-day cortisol infused (B) and 140-day saline-infused (C) fetal lung (200× magnification). Cortisol infusion did not increase the numerical density of SFTP-B-positive alveolar epithelial cells per mm^2^ of lung tissue when compared to the late gestation fetal lung (D). 116-day saline-infused (*n* = 2; open bars), 116-day cortisol-infused (*n* = 4; black bars) and 140-day saline-infused fetuses (*n* = 5; gray bars). Data expressed as mean ± SEM. Data were analyzed by an unpaired Student's *t*-test between 116-day cortisol and 140-day saline groups, *P < 0.05. Scale bar = 50 μm.

The numerical density of SFTP-B-positive AECs in the alveolar epithelium increased with age, but not with cortisol infusion (Fig. 7).

## Discussion

It is widely documented that both endogenous and exogenous GCs regulate genes involved in lung liquid reabsorption and SFTP expression (Tan et al. 1999; Liu et al. 2003; Jesse et al. 2009), processes which are essential for a fetus to make a successful transition to air breathing after birth. However, we have shown that cortisol infusion during the late canalicular phase of lung development in the very preterm sheep fetus does not increase expression of genes involved in these processes to the same degree as occurs after exposure to the normal prepartum surge in cortisol. Fetal intravenous cortisol infusion did, however, increase mRNA expression of *HSD11B-1* within the fetal lung, suggesting that despite a potential increase in the availability of bioactive GC, the very preterm fetal sheep lung is unable to respond to GCs during the late canalicular phase of lung development.

While previous studies have examined the developmental changes or the impact of cortisol infusion between 120- and 130-days gestation on the expression of some genes regulating lung liquid reabsorption in fetal sheep (Liu et al. 2003; Jesse et al. 2009), the relative efficacy of cortisol to mature the very preterm lung in comparison to late gestation has not previously been evaluated. The developmental changes observed in mRNA expression of the *SCNN1-A*, *-B* and *-G* subunits are consistent with those previously reported in sheep (Jesse et al. 2009; Keller-Wood et al. 2009), rats (Tchepichev et al. 1995) and mice (Nakamura et al. 2002). Although the optimal SCNN1 channel subunit combination has yet to be described (Firsov et al. 1998; Kosari et al. 1998; Eskandari et al. 1999), in this study the *SCNN1-B* subunit was the most abundantly expressed in each group, and *SCNN1-A* and *-G* subunits were the second and third most abundantly expressed, respectively. This observed distribution of the expression of subunits in all treatment groups was similar to those observed previously in the lung of the sheep fetus (Jesse et al. 2009).

Water reabsorption is controlled by the differential expression of AQP isoforms in lung tissue and we have observed an increase in mRNA expression of *AQP1*, *3*, and *5*, but not *AQP4* with advancing gestation. The increased *AQP5* and unchanged *AQP4* mRNA expression profiles observed in this study were consistent with previous findings in the fetal sheep lung between 100 and 135-days gestation (Liu et al. 2003). However, AQP expression may be influenced by the composition of the lung tissue studied, for example, there is expression of AQP1 on the vascular endothelium, AQP3 in large airways and to some extent in type II AEC in adults (Kreda et al. 2001), AQP4 in large and small airways and AQP5 in type I AECs (Verkman et al. 2000; King et al. 2004). Unlike the previous study evaluating AQP expression in the fetal sheep (Liu et al. 2003), we have observed an increase in *AQP1* and *3* mRNA expression with advancing gestation. These differences between the two studies may be due to the tissue sampling, therefore, the distribution of components expressing these markers as a result of the heterogeneity of lung tissue, such as expression patterns between smaller and larger airways and alveolar tissue.

In this study, we have demonstrated that high circulating concentrations of cortisol result in increased *HSD11B-1* mRNA expression, suggesting a potential increase in GC availability in the fetal lung. However, despite these findings, there is no change in the expression of genes regulating lung liquid reabsorption, including *SCNN1-A*, *-B*, or *-G* subunits, *ATP1B1*, *AQP1*, *AQP3*, or *AQP5* in the fetal sheep lung in the late canalicular phase of development relative to that which occurs during exposure to the normal prepartum increase in cortisol. It has previously been shown that cortisol infusion at ∼130-days gestation resulted in an increase in only the *SCNN1-A* subunit, but not *SCNN1-B*, *SCNN1-G*, *ATP1A1*, *AQP1*, or *AQP5* mRNA expression in the sheep lung (Jesse et al. 2009). Similarly, maternal dexamethasone treatment from 17 to 19 days gestation (term = 20 days) in rats, during the less mature pseudoglandular phase and early canalicular phase of fetal lung development, enhanced protein expression of the *SCNN1-A* subunit but did not alter *SCNN1-B* or *-G* and *ATP1A1* or *ATP1B1* subunit expression (Tchepichev et al. 1995). In contrast, culture of human fetal lung explants (20–24 weeks gestation) and lung cell lines exposed to dexamethasone exhibited a dose-dependent increase in expression of the *SCNN1 -A*, *-B*, and *-G* subunits (Venkatesh and Katzberg 1997; Itani et al. 2002). The lack of response in the expression of these lung genes to an increase in fetal intravenous cortisol concentrations in this study may be a result of the responsiveness of the fetal sheep lung at 116-days gestation.

In this study, we have demonstrated an increase in gene expression of the catalytic *A1* subunit, but not of the glycolytic *B1* subunit of *ATP1* in response to both cortisol infusion and advancing age. In the fetal rat lung, *ATP1* subunit mRNA expression is altered in response to maternal dexamethasone exposure and gestational age at administration (14- to 19-days gestation) throughout the pseudoglandular and early canalicular stages (Ingbar et al. 1997), which is earlier in lung development than our model. Administration of GC for a shorter duration (1 or 3 days) and earlier ages (14- to 16-days gestation) increases *ATP1B1*, but not *A1* subunit expression, whereas longer treatments (5 days) closer to term (14- to 18-days gestation) increase *ATP1B1* and decrease *A1* subunit expression. Interestingly, closer to term there was no impact of shorter exposure (1 or 3 days) on *ATP1* subunit expression. However, in the rat, type II AEC cultures exposed to dexamethasone exhibit an increase in both *ATP1A1* and *B1* subunit mRNA expression (Barquin et al. 1997). The differing mRNA expression profiles from each study may be due to the developmental stage of the lung, drug type, dose or direct/indirect GC action regulating expression in each species. Despite the increase in *ATP1A1* subunit with cortisol infusion, the findings from this study suggest that in comparison to the level of expression of genes involved in the regulation of lung liquid reabsorption near term, cortisol stimulation around 116 days gestation in the fetal sheep lung is not adequate to increase the expression of genes regulating ion pumps and water channels, which remove lung liquid and contribute to neonatal survival upon premature exposure to the extrauterine environment.

The second important component contributing to the successful transition of a fetus to air breathing at birth involves maturation of the pulmonary surfactant system. The regulation of surfactant proteins by GC is complex and varies as a function of dose and developmental stage (Weaver and Whitsett 1991; Boggaram 2003). The developmental pattern of mRNA expression for *SFTP-A*, *-B* and *-C* observed in this study is similar to previous studies in the sheep fetus (Tan et al. 1999; Flecknoe et al. 2003). Interestingly, lung *SFTP-D* was increased with both age and cortisol infusion. However, the period of cortisol infusion was not adequate to increase *SFTP-A*, *-B* or *-C* mRNA expression to the same extent as those increases in expression observed near term. These findings suggest that despite exposure to increased plasma cortisol concentrations during the late canalicular phase of development, the lung of the very preterm fetus is not responsive to GCs.

GCs stimulate the expression of surfactant proteins in an indirect manner. The promoter regions of the four SFTP genes contain regulatory elements, some of which are conserved between the four genes and others which are not, and hence there is specific regulation of each of the genes. NKX2-1 interacts with various cofactors, such as GATA6 (Bruno et al. 2000; Liu et al. 2002), and binds to NKX2-1 binding elements expressed on the promoter regions of SFTP-A, -B and -C gene constructs (Bruno et al. 1995; Kelly et al. 1996; Mendelson 2000). In the case of SFTP-D, NKX2-1 regulates gene transcription indirectly via interactions with nuclear factor of activated T cells (NFATs) (Davé et al. 2004). Here, we have evaluated gene expression of the transcription factor *NKX2-1*, and its co-factor *GATA6*, and have found that there was no difference in their expression with either age or cortisol infusion. Although there are many different mechanisms by which transcription factors can interact with DNA to influence expression, these data suggest that at the transcriptional level, these two factors may not play a role in surfactant protein expression or GC responsiveness in our cohort.

At ∼116-days gestation, there is differentiation of type I and type II AECs in the fetal sheep lung (Harding and Bocking 2001; Flecknoe et al. 2003). The fact that cortisol infusion from 109- to 116-days gestation did not stimulate greater production of surfactant proteins suggests that there may be a limited capacity of the surfactant system to respond because there are fewer or less mature type II AECs present at this stage of gestation. We found that there was an increase in the number of SFTP-B-positive cells in the alveolar epithelium with age, which is consistent with the increase in *SFTP* mRNA expression. However, there was no impact of cortisol infusion on the number of mature SFTP-B-positive cells in the alveolar epithelium when compared to the number present in the late gestation fetal lung. This result suggests that there is a limitation in the extent to which GCs can induce an increase in number and responsiveness of type II AECs, the cell responsible for an increase in SFTP expression. Taken together, the gene and immunohistochemistry results suggest that even during the last third of gestation, GCs may not be effective if the lung is at the late canalicular stage of development.

In the clinical setting, antenatal GCs are administered to women at risk of preterm birth to promote fetal lung maturation, however, they also have benefits in reducing the risk of other morbidities in preterm infants. In sheep models, it has been demonstrated that both fetal and maternal injections have similar effects on lung development (Rebello et al. 1996). It is widely understood that antenatal GC administration to women at risk of preterm birth from 24 to 37 weeks gestation is the ‘gold standard’ treatment for reducing the risk of respiratory distress syndrome (Roberts and Dalziel 2006) in the 8–12% of babies born preterm in Australia, the UK, and the USA (Laws and Sullivan 2004; Tucker and McGuire 2004; Heron et al. 2010). However, with the primary outcome being to prepare the fetal lung for air breathing, it is important to consider a potential developmental limit to when the lung can respond to such treatments, particularly around the limit of fetal viability.
